# Mapping subcortical brain lesions, behavioral and acoustic analysis for early assessment of subacute stroke patients with dysarthria

**DOI:** 10.3389/fnins.2024.1455085

**Published:** 2025-01-07

**Authors:** Juan Liu, Rukiye Ruzi, Chuyao Jian, Qiuyu Wang, Shuzhi Zhao, Manwa L. Ng, Shaofeng Zhao, Lan Wang, Nan Yan

**Affiliations:** ^1^Guangdong-Hong Kong-Macao Joint Laboratory of Human-Machine Intelligence-Synergy Systems, Shenzhen Institutes of Advanced Technology, Chinese Academy of Sciences, Shenzhen, China; ^2^University of Chinese Academy of Sciences, Beijing, China; ^3^Department of Rehabilitation Medicine, The Eighth Affiliated Hospital of Sun Yat-sen University, Shenzhen, China; ^4^Department of Radiology, The Eighth Affiliated Hospital of Sun Yat-sen University, Shenzhen, China; ^5^Speech Science Laboratory, Faculty of Education, University of Hong Kong, Hong Kong, Hong Kong SAR, China

**Keywords:** subacute stroke, dysarthria, speech production, linguistic processing, articulation, basal ganglia, thalamus

## Abstract

**Introduction:**

Dysarthria is a motor speech disorder frequently associated with subcortical damage. However, the precise roles of the subcortical nuclei, particularly the basal ganglia and thalamus, in the speech production process remain poorly understood.

**Methods:**

The present study aimed to better understand their roles by mapping neuroimaging, behavioral, and speech data obtained from subacute stroke patients with subcortical lesions. Multivariate lesion-symptom mapping and voxel-based morphometry methods were employed to correlate lesions in the basal ganglia and thalamus with speech production, with emphases on linguistic processing and articulation.

**Results:**

The present findings revealed that the left thalamus and putamen are significantly correlated with concept preparation (*r* = 0.64, *p* < 0.01) and word retrieval (*r* = 0.56, *p* < 0.01). As the difficulty of the behavioral tasks increased, the influence of cognitive factors on early linguistic processing gradually intensified. The globus pallidus and caudate nucleus were found to significantly impact the movements of the larynx (*r* = 0.63, *p* < 0.01) and tongue (*r* = 0.59, *p* = 0.01). These insights underscore the complex and interconnected roles of the basal ganglia and thalamus in the intricate processes of speech production. The lateralization and hierarchical organization of each nucleus are crucial to their contributions to these speech functions.

**Discussion:**

The present study provides a nuanced understanding of how lesions in the basal ganglia and thalamus impact various stages of speech production, thereby enhancing our understanding of the subcortical neuromechanisms underlying dysarthria. The findings could also contribute to the identification of multimodal assessment indicators, which could aid in the precise evaluation and personalized treatment of speech impairments.

## 1 Introduction

Dysarthria refers to a motor speech disorder affecting respiration, phonation, resonance, articulation, and prosody (Enderby, 2013; Darley et al., 1969). It is the most common expressive communication deficit in post-stroke patients, with a prevalence of approximately 30–75% (Mackenzie, 2011; Liu et al., 2023; Ali et al., 2015). Recent studies suggested that dysarthria is frequently associated with pathologies in subcortical areas (Enderby, 2013; Rampello et al., 2016; Sperber and Karnath, 2016). For example, Summaka (Summaka et al., 2022) described how damage to the basal ganglia, thalamus, and cerebellum after a stroke led to dysarthria. The subacute phase exhibits distinct pathological characteristics compared to other stages of stroke or other conditions (e.g., Parkinson's disease) (Liu et al., 2023; Koyuncu et al., 2016). This phase is crucial for early and accurate diagnosis and assessment of dysarthria (Mackenzie, 2011; Spencer and Brown, 2018). Although the neural mechanisms underlying dysarthria have garnered significant attention, few studies focused on subcortical damage. Lesions in the basal ganglia and thalamus present different pathological manifestations of speech compared to other regions, such as the motor cortex and the Broca's area (Kearney and Guenther, 2019; Lim et al., 2014; Rampello et al., 2016). Therefore, investigating the impacts of these subcortical structures on speech production is essential for advancing our understanding of the pathological mechanisms of dysarthria following stroke.

Speech production is a complex process that integrates linguistic and motor functions, involving the precise control of muscles responsible for vocalization and articulation, ultimately resulting in the generation of speech signals (Enderby, 2013; Kearney and Guenther, 2019). As outlined by the lexical access model (Wilson et al., 2009), speech is produced through the following stages: conceptual preparation, lexical selection, phonological code retrieval and encoding, and articulation, all of which are influenced by various cerebral and subcortical structures (Levelt, 2000). The early stages of speech production, such as conceptual preparation and word selection, involve processes related to speech or language processing (Liberman and Whalen, 1975; Pennington and Bishop, 2009). Conceptual preparation activates a lexical concept, which links to a lemma - an abstract lexical entry with syntactic but no phonological details. Subsequently, a phonological word form is retrieved and encoded through levels: phonological, then phonetic, leading to a motor program for articulation (Wilson et al., 2009; Levelt et al., 1999; Levelt, 2000). In recent years, many studies have revealed that the basal ganglia are associated with the early stages of speech processing outlined above (Guenther, 2007; Brown and Marsden, 1998; Graybiel, 2000), specifically, the basal ganglia play a critical role in initiating and organizing linguistic concepts and in selecting and retrieving words from the mental lexicon (Duffy et al., 2012; Silveri, 2021). Lesions in this region can result in anomia, characterized by difficulty in retrieving specific words or names (Camerino et al., 2024; Magee et al., 2019).

The stages of linguistic processing stages are succeeded by the phonological encoding and articulation phases, controlled by a coordinated set of motor activities involving the respiratory, laryngeal, and articulatory systems, which are critical for producing different speech sounds (Wilson et al., 2009). Currently, the DIVA model is the most widely accepted neural network model for elucidating the neural and motor processes involved in speech production (Kearney and Guenther, 2019). Its input corresponds to the output of the phonological encoding stage, and it provides detailed neural and computational explanations of both the phonological encoding and articulation stages(Wilson et al., 2009), as well as the neural processes carried out by specific neuroanatomical regions (e.g., motor cortex, basal ganglia, thalamus) (Guenther, 2007). For instance, the DIVA model suggests that damage to the basal ganglia can disrupt both feedforward and feedback systems, leading to dyskinesia of the vocal organs (Tourville and Guenther, 2011; Guenther, 2007). Studies have shown that the basal ganglia are crucial for the planning and execution of fluent speech motor sequences, ensuring the precise timing and coordination of articulatory muscle movements (Duffy et al., 2012; Silveri, 2021). They also aid in the selection and initiation of speech-motor programming (Kempler and Van Lancker, 2002). Lesions to the basal ganglia can result in dysarthria, characterized by slurred speech, imprecise articulation, and irregularities in pitch, volume, and rhythm (Guenther, 2007), ultimately impairing speech muscle control and reducing vocal clarity (Silveri, 2021).

The thalamus serves as a critical conduit between subcortical nuclei and the cerebral cortex, playing a key role in speech and motor functions, with pathologies frequently leading to dysarthria (Graybiel, 2000; Bostan and Strick, 2018). Studies have demonstrated that the thalamus is essential for conceptual preparation, coordinating the integration of sensory and cognitive information (Pennington and Bishop, 2009). Its extensive connections with other brain regions enable the integration of diverse inputs, thereby influencing decision-making during lexical selection (Pickett et al., 1998). Thalamus also supports the fluency and consistency of phonological encoding processes (Wallesch et al., 1983). However, speech impairments resulting from thalamic pathologies, such as strokes and Parkinson's disease, are often overlooked or misdiagnosed as cognitive or motor deficits (Camerino et al., 2024), leaving the specific contributions of the thalamus to speech production insufficiently understood (Guenther, 2007; Johnson and Ojemann, 2000). Stroke patients with basal ganglia or thalamic damage frequently exhibit mild cognitive decline, complicating efforts to fully isolate the influence of cognitive factors - an enduring limitation in prior research (Liberman and Whalen, 1975).

To further explore the neuromechanisms of speech processing, researchers have designed a series of classic speech behavioral experimental tasks, including speech fluency tasks and picture-naming tasks (Levelt and Meyer, 2000; Kempler and Van Lancker, 2002). Recent research has integrated neurophysiological and behavioral approaches, offering a comprehensive view of speech-related brain functions and their impairments (Chow et al., 2023). For articulation, the Frenchay Dysarthria Assessment (FDA) is a widely used clinical tool for diagnosing and assessing dysarthria, as well as the motor function of each vocal organ (Enderby, 1980). Additionally, acoustic analysis has been utilized to quantify the variability of pathological speech (Paja and Falk, 2012). However, studies describing speech impairments often conflate measures of speech processing and articulation (Gagnon et al., 2018), leading to an incomplete understanding of the distinct contributions of each nucleus within the basal ganglia and thalamus to speech functionality, vocal movement, and speech quality (Nambu, 2008).

To address these issues, we first focused on subacute stroke patients with basal ganglia or thalamic damage and attempted to clarify the relationship between subcortical mechanisms and speech impairments by integrating neuroimaging, behavioral, and acoustic analyses. Voxel-Based Morphometry (VBM) and Voxel-Based Lesion-Symptom Mapping (VLSM) techniques were employed to precisely quantify the location and extent of these lesions (Bates et al., 2003). A series of speech tasks assessing fluency, picture association, naming, and color identification were administered to evaluate the linguistic functionality of speech production. Cognitive ability was also measured as an independent variable to determine the direct and independent effects of basal ganglia or thalamic lesion. The findings enhance our understanding of the neural basis of dysarthria, help identify multimodal potential biomarkers, and may lead to more precise and personalized treatments for speech impairments.

## 2 Materials and methods

### 2.1 Participants

All data were obtained from 42 participants, including 20 subacute stroke patients and 22 healthy controls. Patient recruitment followed specific inclusion and exclusion criteria. Inclusion criteria were as follows: (1) subacute stroke patients (first stroke within 6 months); (2) diagnosis of dysarthria; (3) subcortical damage; (4) age over 18; (5) at least a primary school education; (6) no prior speech or language therapy. Exclusion criteria included: (1) vision or hearing impairment; (2) dementia or psychiatric disorders; (3) other neurological conditions unrelated to the stroke. Based on demographic information of patients, 22 healthy individuals were selected as the control group. Detailed participant information is presented in Table 1. This study was approved by the Shenzhen Institute of Advanced Technology and the Eighth Affiliated Hospital of Sun Yat-sen University (Ethics Code: SIAT-IRB-220415-H0598). Written informed consent was obtained from all participants or their legal representatives prior to data collection.

**Table 1:** Demographic information and clinical evaluation results of all subjects.

	**Patient**	**Normal**	**Statistic (t/p)**
Number(*N*)	20	22	42
Gender(M:F)	16:4	16:6	0.58/0.30
Age(year)	59.65(14.02)	60.68(8.79)	−0.28/0.77
Education(year)	10.95(3.75)	11.82(3.76)	−0.74/0.45
MoCA(30)	20.90(5.22)	27.48(2.09)	−5.45^**^/0.00
FDA(0-4)	3.24(0.47)	4.00(0.00)	−7.42^**^/0.00

The statistics for Gender are χ^2^ and p values (Male, M; Female, F). ^*^indicates *p*-value, and the following is detailed representation: ^*^p < 0.05, ^**^p < 0.01, ^***^p < 0.001.

All patients underwent a thorough clinical evaluation by certified Speech Language Therapists (SLTs) and Neurologists. Cognitive and speech ability of participants were assessed using the Montreal Cognitive Assessment (MoCA) (Nasreddine et al., 2005) and the Frenchay Dysarthria Assessment (FDA) (Enderby, 1980; Ghio et al., 2019), respectively. The FDA evaluates 28 distinct dimensions of speech production, categorized into eight aspects: reflex, respiration, lips, jaw, velum, laryngeal, tongue, and intelligibility. The intelligibility category comprises word, sentence, conversation, and speed. Each dimension was rated on a scale of 0 (no function) to 4 (normal function). For more detailed FDA scores of all patients, refer to Supplementary Table 2.

### 2.2 Data collection

#### 2.2.1 Speech data

Speech materials were designed with consideration of the phonetic features of Chinese and the diverse impairment profiles observed in dysarthria, including syllables and characters. (1) Syllables: a total of 200 distinct syllables (monosyllabic, disyllabic, and multisyllabic) were included, covering high-frequency syllables, low-frequency syllables, and easily confused syllables. (2) Characters: a total of 90 high-frequency characters were selected, including numbers, direction, and basic verbs. Audio signals were recorded in a soundproof room using a professional-grade microphone (Takstar, MS400), with a sampling rate of 16 kHz, 16-bit encoding, and single-channel recording. The microphone was positioned approximately 8-10 cm from the speaker's mouth. Further details of each task and the experimental paradigm can be found in previous studies (Liu et al., 2023).

#### 2.2.2 Behavioral data

**Figure 1:**
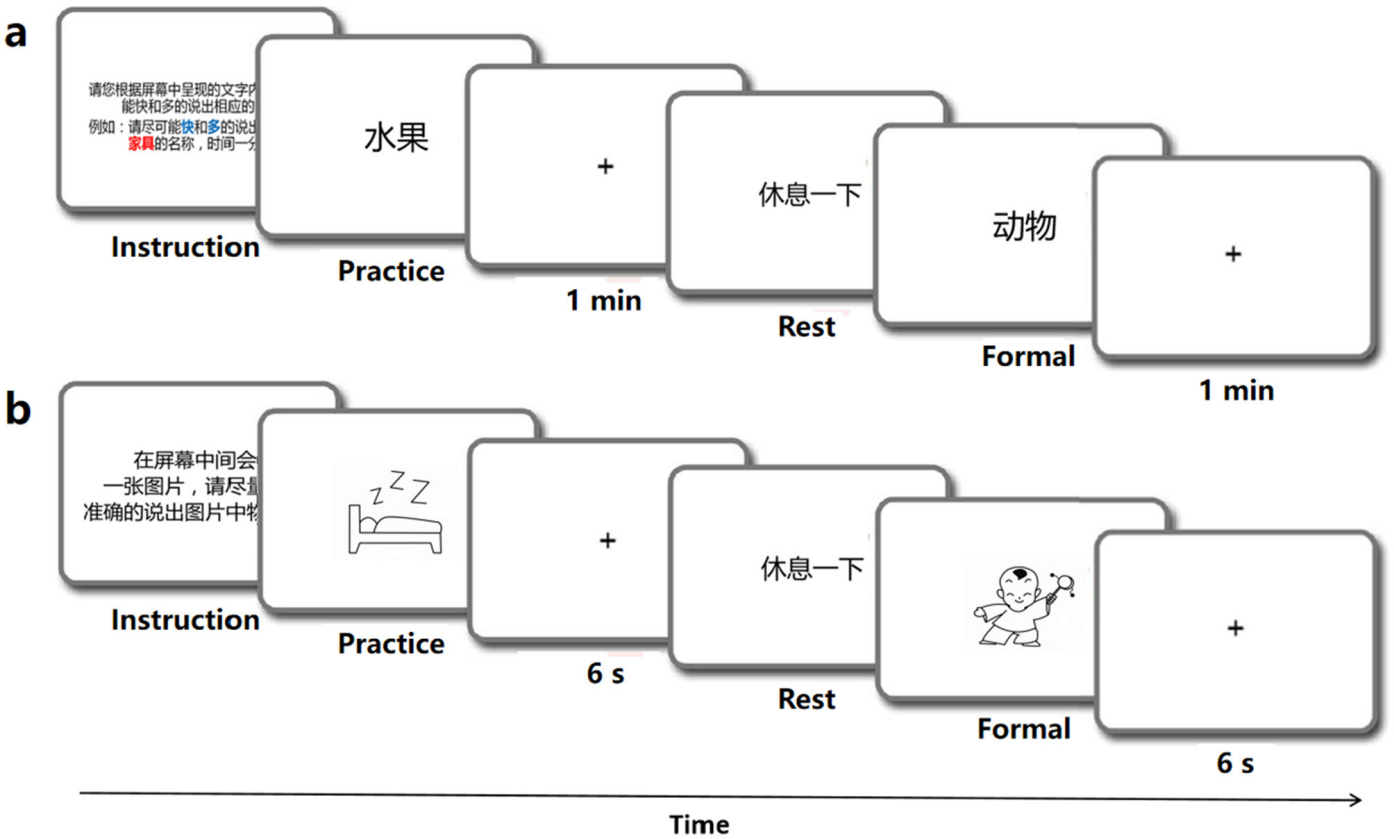
Schematic diagram of the behavior experiments program.

The linguistic processing of speech production was evaluated by using four classic tasks, which incolving the stages of conceptual preparation and lexical selection (Levelt, 1992; Indefrey and Levelt, 2004). The tasks included a speech fluency task (Wang et al., 2019), a picture association task (Lin et al., 2020), a picture naming task (Liu et al., 2011), and a color naming task (Monsell et al., 2001). Each behavioral task involved common objects and was conducted in separate sessions using the E-prime program and Chronos system (Babjack et al., 2015) for precise measurement of reaction times of sound onset on each trial. The order of item presentation was randomized for each task and consistent across subjects. There was a 1-minute response limit for the speech fluency task and a 6-second limit for the other tasks. Testing was conducted individually in a quiet room, with each session lasting for no more than half an hour, including rest breaks as needed. The schematic diagram of the behavior experiment procedure is shown in Figure 1. In addition to recording the reaction time of each behavioral task, synchronized audio data were also obtained using Praat software (Boersma, 2001). The behavioral audio data were recorded using the same equipment as the speech data mentioned above.

Speech fluency task (SF). The SF task was designed to examine linguistic functioning and speech fluency (Wang et al., 2019). In the SF task, participants were instructed to produce as many words as they could within one minute which were under each of the ten semantic categories such as fruits, animals, vehicles, etc. A practice block was provided with a different category (weather) to familiarize participants with the task, but was not included in the analysis (see Figure 1A). To avoid potential memory effects, no further instructions were given during the experimental trials.

Picture association task (PA). Following Lin et al. (2020), this task involved naming randomly presented black-and-white outlined drawings on a screen. The stimuli consisted of 40 black-and-white outline drawings displayed on a white background. Each trial was displayed for 6 seconds, following a 50 ms beep (see Figure 1B). Meanwhile, participants were expected to verbally name the picture. The 40 stimuli were randomly arranged in a sequence, with 10 trials comprising a block. Participants were trained to name six additional pictures aloud before the experiment to ensure readiness for the formal task. The task was repeated to enhance the quality of the behavioral data.

Picture naming task (PN). This task followed the same format as the PA task, and the stimulus material was based on Liu et al. (2011). A total of 100 items were selected from all 435 object line drawings, with the concept familiarity, subjective word frequency, image agreement, image variability, and visual complexity of these pictures balanced. Participants were instructed to name each picture quickly and accurately.

Color naming task (CN). This task followed the same format as the PA and PN tasks (see Figure 1B). A total of 36 most common object words were selected as experimental stimuli, all of which possessed obvious color characteristics, such as banana and milk. Participants were instructed to quickly and accurately name the color of each item (Monsell et al., 2001).

#### 2.2.3 Imaging data

The imaging data of the participants were acquired from the Eighth Affiliated Hospital of Sun Yat-sen University using a 3T Siemens scanner. We collected three types of images: (i) high-resolution 3D T1-weighted images; (ii) FLAIR T2-weighted images; and (iii) diffusion-weighted images. The 3D images were T1- weighted 3D MPRAGE images on the sagittal plane with parameters: matrix size = 512 × 512, voxel size = 0.5 × 0.5 × 0.5 *mm*^3^, repetition time = 12 ms, echo time = 4.2 ms, inversion time = 400 ms, field of view = 256 × 256 *mm*^2^, flip angle = 15°, slice number = 320 slices. The FLAIR T2 images were FLAIR T2-weighted images on the axial plane with parameters: matrix size = 512 × 512, voxel size = 0.5 × 0.5 × 5 *mm*^3^, repetition time = 8000 ms, echo time = 122 ms, inversion = 2 s, field of view = 250 × 250 *mm*^2^, flip angle = 90°, slice number = 28 slices. Diffusion-weighted imaging had two separate sequences with different diffusion weighting direction sets so 32 directions were covered in total. The first acquisition had the following parameters: 12 diffusion weighting directions, matrix size = 128 × 128, voxel size = 2.0 × 2.0 × 2.6 *mm*^3^, repetition time = 13 000 ms, echo time = 69 ms, inversion time = 0 s, field of view = 250 × 250 *mm*^2^, flip angle = 90°, slice number = 53 slices. The other acquisition had the same parameters except that it included 20 different directions. The first two volumes were *b*_0_ volumes and the b-value of other volumes was 1,000 s/*mm*^2^ in each sequence.

### 2.3 Data analysis

#### 2.3.1 Speech data annotation and acoustic feature extraction

All the speech data were segmented and transcribed verbatim by two experienced researchers using Praat (Boersma, 2001). Relevant speech materials were manually transcribed in Chinese characters on the first tier. Subsequently, the speech content of each participant was transcribed in Pinyin (a phonetic representation of Chinese) on the second tier. The character and syllable tasks were manually segmented into vowel and consonant segments, which were marked on the third tier based on their spectrograms and auditory judgments. The method for annotating vowels and consonants is presented in Supplementary Figure 1.

Since vowel distortion and consonant errors are characteristic manifestations of dysarthria, acoustic features were extracted from the vowel segments. A comprehensive 12-dimensional set of acoustic features was derived from the medial 80% of the steady-state vowel segment, encompassing the following parameters: vowel duration, F1 and F2 variability, jitter, shimmer (Hernandez et al., 2020), harmonics-to-noise ratio (HNR) (Karan et al., 2020), tongue distance, jaw distance (Sapir et al., 2010), Vowel Space Area (VSA), Formant Centralization Ratio (FCR) (Banks et al., 2019), degree of movement (F2i/F2u ratio) (GE et al., 2021), and Vowel Articulation Index (VAI).

#### 2.3.2 Behavioral data annotation and feature extraction

To ensure the precise calculation of both the number of valid responses and the reaction times for each behavioral task, all audio data underwent manual annotation. For the SF task, correct answers were annotated within one minute and counted within each 15-s time window, including four periods: 0–15 s (T1), 15–30 s (T2), 30–45 s (T3), and 45–60 s (T4). For the PA, PN, and CN tasks, each “beep” voice and effective answer were also labeled on the first tier. The reaction time was determined by the interval between the “beep” and the response, as depicted in Supplementary Figure 2.

Meanwhile, behavioral features were extracted corresponding to different tasks. For the SF task, SF_T1, SF_T2, SF_T3, and SF_T4 represented the number of words within 0–15 s, 15–30 s, 30–45 s, and 45–60 s windows. Reaction times (RT) for each trial in the PA, PN, and CN tasks were also directly extracted for each subject.

#### 2.3.3 Imaging data preprocessing

All DICOM files were converted into NifTi format using SPM12 software, and the data quality, including sharpness, whole-head coverage, and orientation, was carefully checked. We first coregistered the two T1-weighted structural imaging data in the same native space using the trilinear interpolation method implemented in SPM12, resulting in an averaged structural image. Subsequently, we coregistered and resliced the FLAIR T2 images to the averaged structural image using the same trilinear interpolation method in SPM12. The lesion contours of each patient were drawn on the T1 structural image by one trained person, slice-by-slice, visually referring to the FLAIR T2 images in FSL (http://fsl.fmrib.ox.ac.uk). These lesion boundaries were further validated by a neuroradiologist (Q.W.) from the Eighth Affiliated Hospital of Sun Yat-sen University, utilizing T2 and DWI images. Patients with diffuse damage or cortical lesions that could not be accurately bounded were excluded from the study. Then, the structural images were resliced into 1 × 1 × 1*mm*^3^ voxel sizes.

For normalization, a manual registration technique was applied to minimize the impact of lesions on brain image distortion. This approach has been documented by Li et al. (2017) and Zhao et al. (2017). Structural images from each patient were manually registered into Talairach space using the “3D Volume Tools” in BrainVoyager QX v2.0 (www.brainvoyager.com). ANTs software was utilized to estimate the affine transformation matrix between the native and Talairach spaces. Using this matrix, we then transformed the lesion maps into Talairach space using the “WarpImageMultiTransform” program. Finally, the lesion maps were further transformed into MNI space for the next analysis.

#### 2.3.4 Lesion-symptom mapping analysis

The present study utilized structural MRI to evaluate brain damage severity through two primary indices: the lesion status, expressed as a lesion percent value, and the volume of gray matter (VGM value). The lesion percent is calculated as the number of voxels with lesions divided by the total number of voxels in each area. Consistent with the methodology of Han et al. (2013), identical parameters for scanning and preprocessing were used to generate the lesion map. The lesion index reflects physical damage to voxels, encompassing both white and gray matter. It serves as a dichotomous variable, classifying each voxel as either intact or lesioned, and is widely regarded as a classical indicator of lesion severity (Meyer et al., 2016). In the present study, each voxel in every patient was assigned a lesion value (categorical variable) derived from the lesion map, along with a structure volume (continuous variable). It is noted from prior research that brain regions with lesions often show reduced VGM values compared to undamaged areas (Fox et al., 2016), suggesting a correlation between these variables.

#### 2.3.5 Voxel-based morphological analysis

Structural image analysis was performed using SPM12 software. The technical details of primary cortical reconstruction and volumetric segmentation procedures have been previously described (Chen et al., 2020; Weisstein, 2004). In brief, the processing included removal of nonbrain tissue using a hybrid watershed/surface deformation procedure, automated Talairach transformation, segmentation of the subcortical white matter and deep gray matter volumetric structures, intensity normalization, tessellation of the gray matter/white matter boundary, automated topology correction, and surface deformation following intensity gradients to optimally place the gray/white and gray/cerebrospinal fluid (CSF) borders at the location where the greatest shift in intensity defined the transition to the other tissue class. Cortical thickness was calculated as the closest distance from the gray/white matter boundary to the gray/ CSF boundary at each vertex. Adjustments were then made for differences in head size and volumes for each region (white matter and subcortical structure volume) were adjusted to intracranial volume (ICV).

#### 2.3.6 Statistical analysis

First, statistical analyses were conducted on behavioral and acoustic features to assess speech impairments. A linear mixed-effects regression model (Liu et al., 2023) was employed to investigate the differences in linguistic processing and speech quality between the dysarthria and control groups. Each model incorporated fixed effects for the group (dysarthria vs. normal), cognitive level as measured by Montreal Cognitive Assessment (MoCA) scores, and participants and age as random intercepts. The slope estimates of the model, which indicate the average impact of dysarthria or cognitive status on speech, are expressed as beta coefficients (B) with standard errors (SE). A positive B value suggests an increase in the measured parameter, while a negative value suggests a decrease.

To ascertain the direct impact of subcortical nuclei on articulatory movement and linguistic functions, the Pearson correlation analysis was conducted separately for each acoustic feature, behavioral feature, FDA score, and VGM. This included comparisons between specific acoustic features (e.g., tongue distance, Vowel Space Area [VSA]), FDA scores, and behavioral features (e.g., Speech Fluency task at 0–15 s [SF_T1], Reaction Time of PA task [RT_PA]), as well as the volume of gray matter (VGM) and the percentage of brain lesions in the basal ganglia and thalamus. Correlation coefficients were calculated between each objective acoustic-behavioral feature, subjective FDA scores, and the VGM, as well as the lesion percentages. A Bonferroni correction was applied to adjust for multiple comparisons (Weisstein, 2004), with the threshold for statistical significance set at an alpha level of 0.05. Statistical models were developed using the R programming language and the lme4 package (Bates, 2010).

## 3 Results

### 3.1 Distribution and proportion of subcortical structure damage

**Figure 2:**
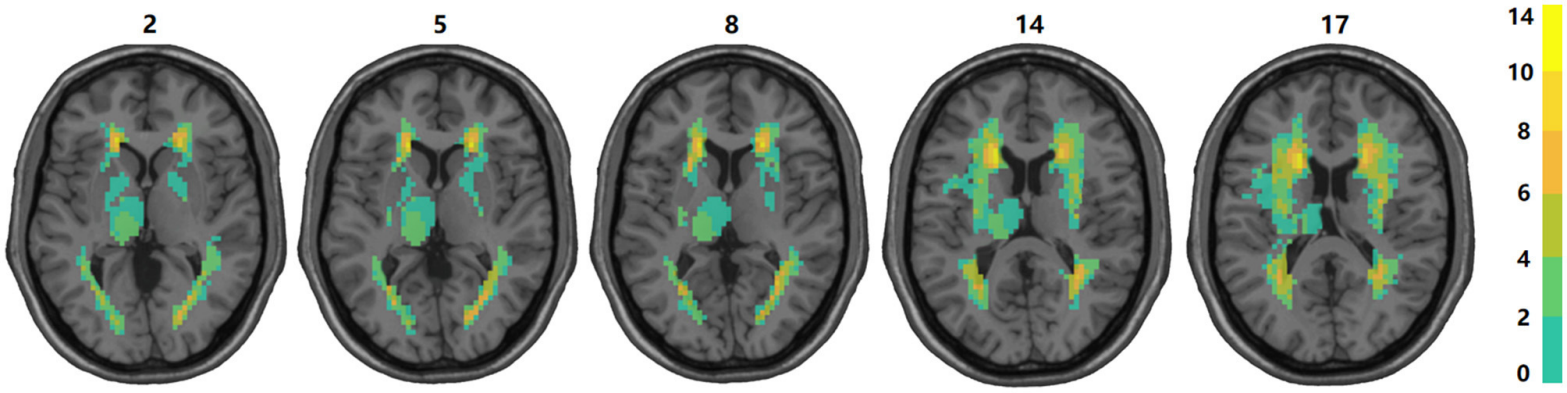
Overlap of lesions in our sample of participants. The color bar indicates the number of participants having a lesion at a given location. The upper boundary (*n* = 14) of the color scale represents the highest lesion overlap among the 20 participants included in the final data analyses. The values above the slices indicate the z coordinates in the Montreal Neurological Institute space. In this figure, left is left.

Under the guidance of professional radiologists, the precise extent and boundaries of brain lesions for each patient were carefully outlined. The distribution and proportion of lesion locations within subcortical nuclei were then assessed. The distribution of subcortical lesions in the study cohort is illustrated by the lesion prevalence map shown in Figure 2. The subsequent analysis focused on subcortical nuclei where the damage ratio surpassed 20% in all patients (at least 4 patients) to ensure the statistical validity and reliability of our findings. This included an evaluation of the caudate nucleus, putamen, globus pallidus, and thalamus. For more detailed lesion information of the 20 subacute stroke patients, refer to Supplementary Tables 1, 7.

### 3.2 Behavioral and acoustic results

**Figure 3:**
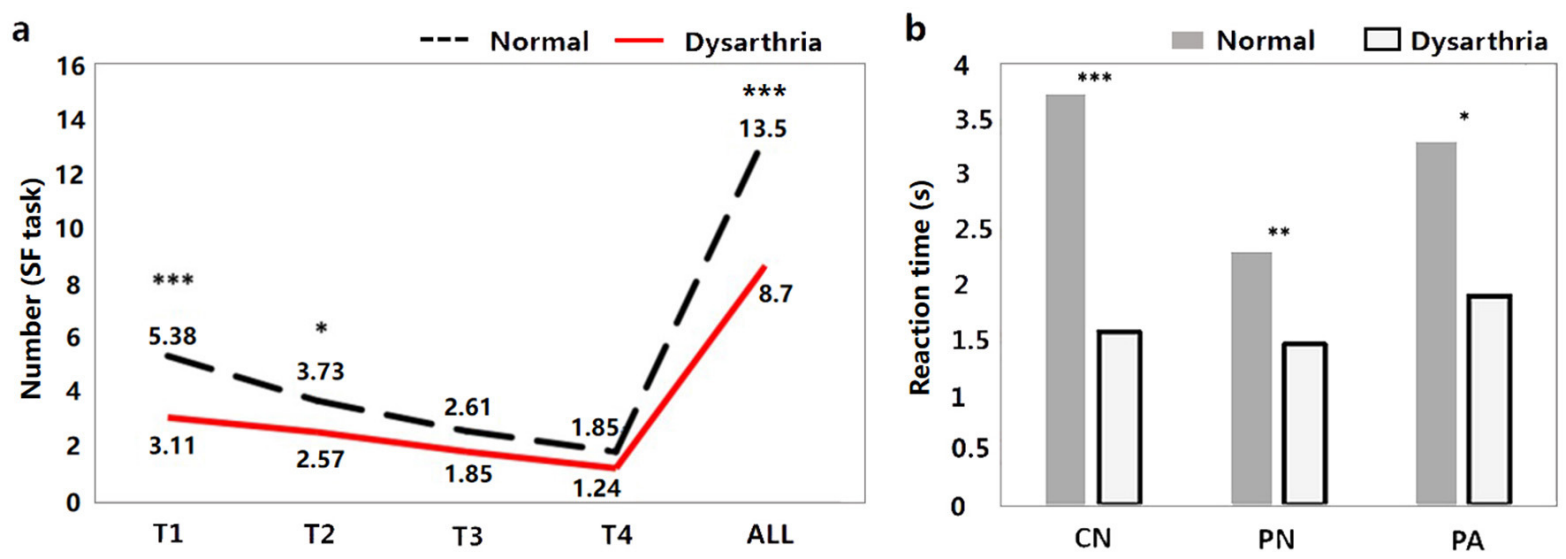
The behavioral results between dysarthria and normal groups (^*^p < 0.05, ^**^p < 0.01, ^***^p < 0.001).

The results of the behavioral tasks reflect the significant differences on linguistic processing between the dysarthric and normal group. As shown in Figure 3A, there are significant differences in the T1, T2, and ALL windows of SF task between dysarthria and normal groups (*t* = *4.68, p* < * 0.001; t* = *2.11, p* = *0.03, t* = *3.71, p* < *0.001*). Figure 3B suggests that the RTs of CN, PN, and PA tasks of patients were longer than normal controls (*t* = *-7.13, p* < *0.001; t* = *-3.19, p* = *0.001; t* = *-3.60, p* < *0.001*), and the significance of differences between two groups gradually diminishes with the difficulty of tasks. In addition, comparing to the influence of cognition, we found significant differences in the T1, T2, T3, T4, and ALL windows (*t* = *2.59, p* = *0.009; t* = *2.29, p* = *0.02; t* = *2.92, p* = *0.003; t* = *2.53, p* = *0.011; t* = *2.39, p* = *0.016*). The detailed statistical results of behavioral features are presented in Supplementary Tables 2, 3.

**Figure 4:**
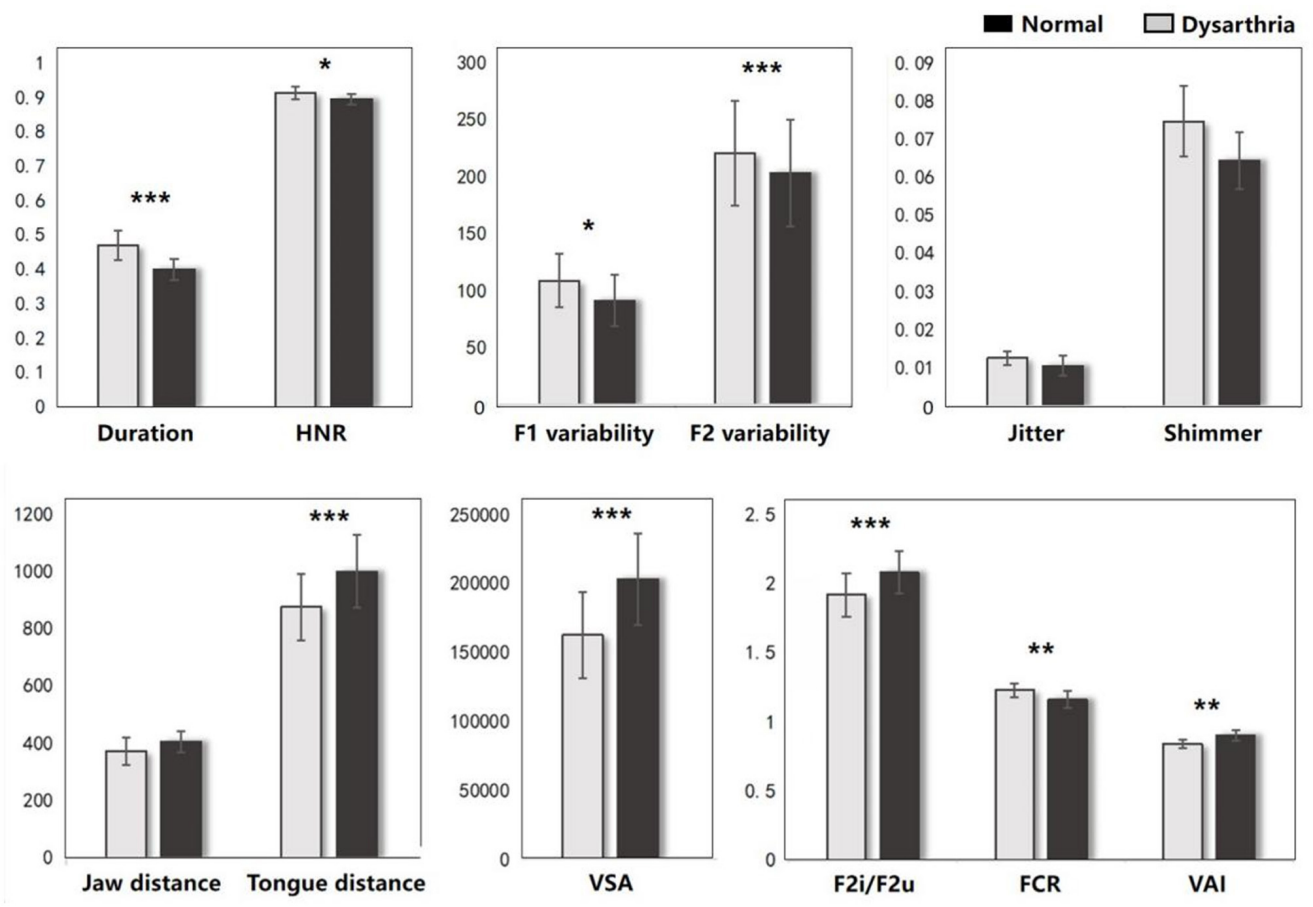
The acoustic results between dysarthria and normal groups (^*^p < 0.05, ^**^p < 0.01, ^***^p < 0.001).

The FDA and acoustic results represent the significant difference on articulation stage between the dysarthric and normal group. As shown in Figure 4, dysarthria patients had significantly longer vowel durations (*t* = *6.81, p* < *0.001*), higher F1/2 variability vs. controls (*t* = *-2.74, p* = *0.005; t* = *-8.43, p*<*0.001*). In voice quality, patients had higher jitter, shimmer, and HNR (*t* = *0.77, p* = *0.43; t* = *1.50, p* < *0.13; t* = *2.02, p* = *0.04*). In articulation, patients showed significantly lower jaw distance, tongue distance, and movement degree (*t* = *1.74, p* = *0.08; t* = *4.22, p* < *0.001; t* = *3.43, p* < *0.001*). In vowel space measures, patients had significantly lower vowel space area (VSA) and vowel articulation index (VAI), and higher vowel centralization (FCR) than controls (*t* = *4.60, p* < *.001; t* = *3.14, p* = *0.001; t* = *-2.91, p* = *0.003*). In addition, when comparing the impact of cognitive factors, significant differences were observed only in vowel duration and F2 variability between dysarthria patients and normal controls (*t* = *-5.02, p* < *0.001; t* = *3.03, p* = *0.002*). The complete statistical values of acoustic features are referred in Supplementary Table 1.

### 3.3 The lesion-behavioral-acoustic results of basal ganglia

#### 3.3.1 Caudate nucleus

**Figure 5:**
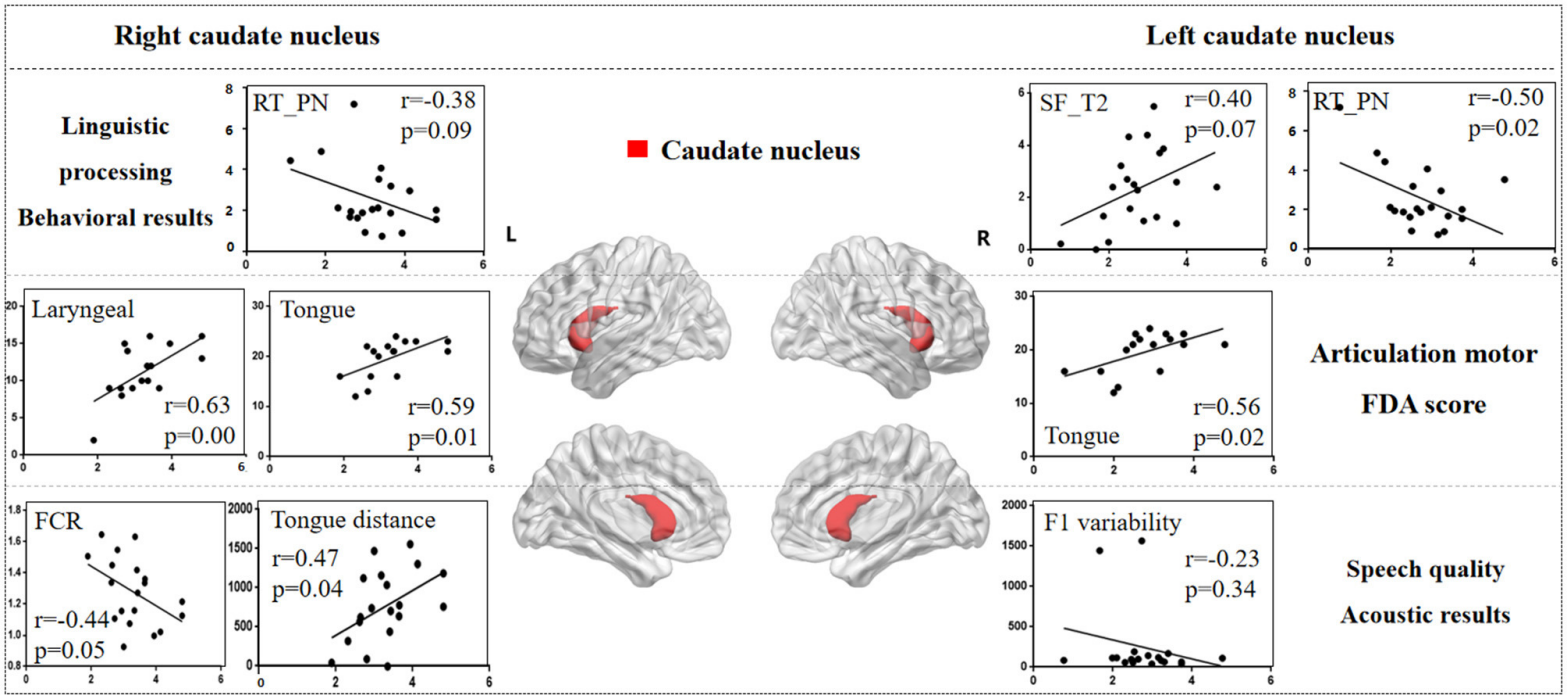
The correlation results between caudate nucleus and behavioral tasks, FDA scores, acoustic features. The x-axis represents the gray matter volume (VGM) of the left or right caudate nucleus, while the y-axis displays the values of various behavioral and acoustic parameters, as well as the FDA scores.

Figure 5 illustrates the correlation results between the caudate nucleus and various aspects of the speech production process, including each speech behavioral task (SF, PA, PN, CN tasks), the motor ability of each vocal organ (the FDA score of each category), and acoustic features (e.g., vowel duration, jaw distance, VSA). The detailed results associated with the caudate nucleus are presented in Supplementary Table 4.

Left caudate nucleus results. (1) Behavioral tasks: there were significant correlations observed between damage to the left caudate nucleus and the SF_T2 window, PN task (*r* = *0.40, p* = *0.07; r* = *-0.50, p* = *0.02*), as well as a moderate correlation with the SF_T3 window and PA task (*r* = *0.30, p* = *0.19; r* = *-0.39, p* = *0.08*). (2) FDA score of each category: there were significant correlations observed between damage to the left caudate nucleus and the Tongue (*r* = *0.56, p* = *0.02*), as well as a mild correlation with the Laryngeal and the speed of intelligibility (*r* = *0.24, p* = *0.35; r* = *0.22, p* = *0.40*). (3) Acoustic features: there were mild correlations between the left caudate nucleus and the vowel duration, F1/F2 variability (*r* = *-0.22, p* = *0.35; r* = *-0.23, p* = *0.34; r* = *-0.20, p* = *0.39*).

Right caudate nucleus results. (1) FDA score of each category: significant correlations have been observed between the right caudate nucleus and the Laryngeal, Tongue, word and speed of intelligibility (*r* = *0.63, p* = *0.008; r* = *0.59, p* = *0.01; r* = *0.61, p* = *0.01; r* = *0.56, p* = *0.02*), and moderate correlations with the Lips, sentence and conversation intelligibility (*r* = *0.46, p* = *0.06; r* = *0.42, p* = *0.10; r* = *0.42, p* = *0.10*). (2) Behavioral tasks: there were mild correlations observed between the right caudate nucleus and the PN, PA tasks, and SF_T2 window (*r* = *-0.38, p* = *0.09; r* = *-0.19, p* = *0.41; r* = *0.19, p* = *0.40*). (3) Acoustic features: meanwhile, there also were mild correlations between the right caudate nucleus and the Duration, F1/2 variability (*r* = *-0.22, p* = *0.35; r* = *-0.23, p* = *0.34; r* = *-0.20, p* = *0.39*).

#### 3.3.2 Putamen

**Figure 6:**
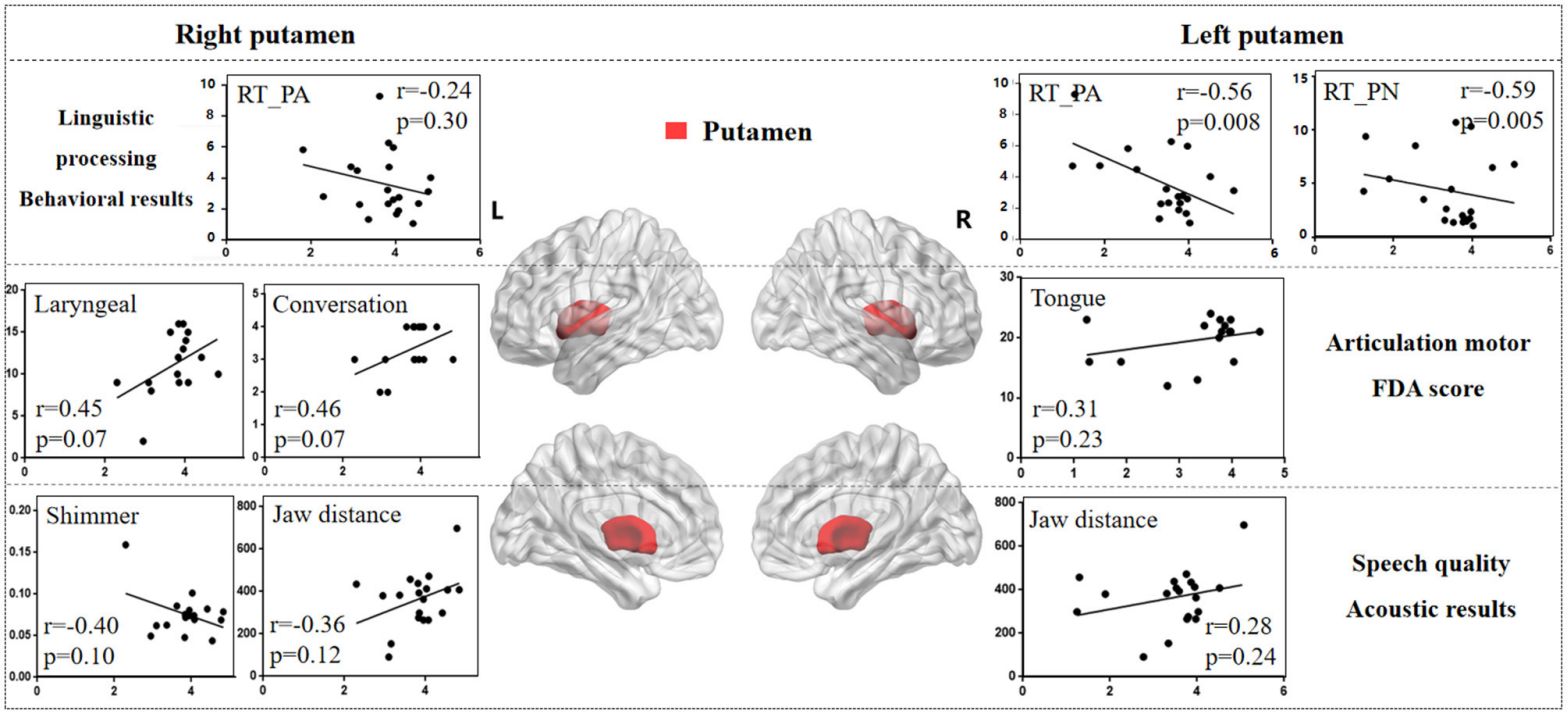
The correlation results between putamen and behavioral tasks, FDA scores, acoustic features. The x-axis represents the gray matter volume (VGM) of the left or right putamen, while the y-axis displays the values of various behavioral and acoustic parameters, as well as the FDA scores.

Figure 6 illustrates the correlation results between the putamen and various aspects of speech production. The detailed putamen results are presented in Supplementary Table 5.

Left putamen results. (1) Behavioral tasks: there were significant correlations observed between damage to the left putamen and the PA, PN tasks, SF_T2 window (*r* = *-0.56, p* = *0.008; r* = *-0.59, p* = *0.005; r* = *0.47, p* = *0.03*), as well as a moderate correlation with the SF_T3 window and CN task (*r* = *0.38, p* = *0.09; r* = *-0.21, p* = *0.37*). (2) FDA score of each category: there were only mild correlations observed between left putamen and Laryngeal, Tongue (*r* = *0.31, p* = *0.23; r* = *0.31, p* = *0.23*). (3) Acoustic features: there were also mild correlations between left putamen and vowel duration, F1/F2 variability (*r* = *-0.24, p* = *0.32; r* = *-0.27, p* = *0.26; r* = *-0.27, p* = *0.25*).

Right putamen results. (1) FDA score of each category: significant correlations have been observed between the right putamen and the Laryngeal, conversation intelligibility (*r* = *0.45, p* = *0.07; r* = *0.46, p* = *0.07*), and mild correlations with the Respiration, Jaw, Tongue, and speed of intelligibility (*r* = *-0.30, p* = *0.24; r* = *-0.23, p* = *0.37; r* = *0.33, p* = *0.20; r* = *0.38, p* = *0.14*). (2) Acoustic features: meanwhile, there were moderate correlations between the right putamen and the Shimmer, Jaw distance (*r* = *-0.40, p* = *0.10; r* = *0.36, p* = *0.12*), and mild correlations with the Jitter, HNR, VSA (*r* = *-0.29, p* = *0.25; r* = *0.20, p* = *0.41; r* = *0.27, p* = *0.26*). (3) Behavioral tasks: there were only mild correlations observed between right putamen and the PA, PN tasks (*r* = *-0.24, p* = *0.30; r* = *-0.21, p* = *0.36*).

#### 3.3.3 Globus pallidus

**Figure 7:**
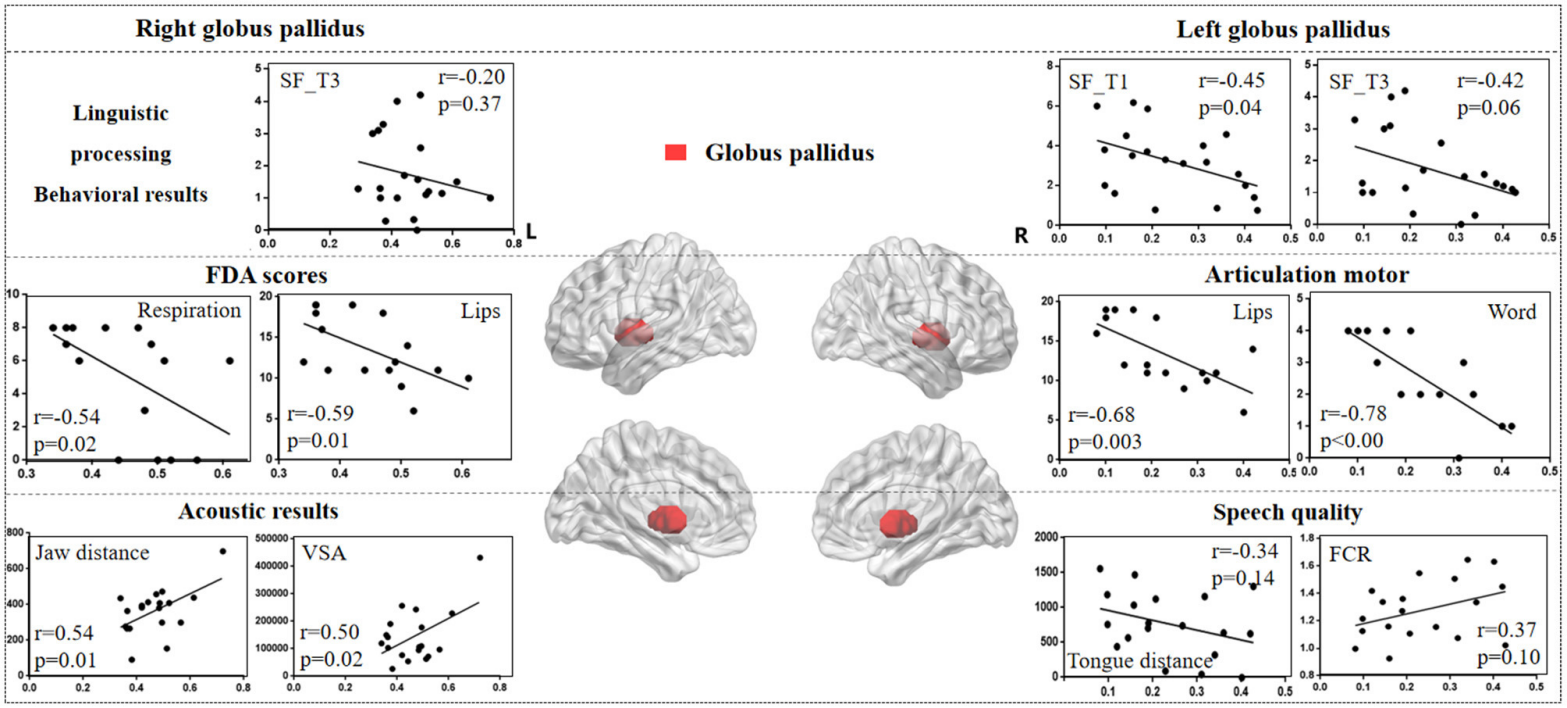
The correlation results between globus pallidus and behavioral tasks, FDA scores, and acoustic features. The x-axis represents the gray matter volume (VGM) of the left or right globus pallidus, while the y-axis displays the values of various behavioral and acoustic parameters, as well as the FDA scores.

Figure 7 illustrates the correlation results between the globus pallidus and various aspects of speech production. The detailed globus pallidus results are presented in Supplementary Table 6.

Left globus pallidus results. (1) FDA score of each category: there were significant correlations observed between damage to the left globus pallidus and the Respiration, Lips, Laryngeal, Tongue, and word, sentence, conversation intelligibility, and speed of intelligibility (*r* = *-0.48, p* = *0.05; r* = *-0.68, p* = *0.003; r* = *-0.63, p* = *0.008; r* = *-0.60, p* = *0.01; r* = *-0.78, p* < *0.001; r* = *-0.52, p* = *0.03; r* = *-0.52, p* = *0.03; r* = *-0.59, p* = *0.01*), as well as a moderate correlation with the Jaw (*r* = *-0.32, p* = *0.22*). (2) Behavioral tasks: there were significant correlations between left globus pallidus and SF_T1 and T3 windows (*r* = *-0.45, p* = *0.04; r* = *-0.42, p* = *0.06*), and moderate correlations with SF_T2 and T4 windows, and PN task (*r* = *-0.33, p* = *0.15; r* = *-0.22, p* = *0.34; r* = *0.23, p* = *0.31*). (3) Acoustic features: there were only mild correlations between left globus pallidus and vowel duration, F1/F2 variability, Tongue distance, F2i/F2u, FCR, and VAI (*r* = *0.30, p* = *0.21; r* = *0.31, p* = *0.19; r* = *0.30, p* = *0.20; r* = *-0.34, p* = *0.14; r* = *-0.31, p* = *0.18; r* = *0.37, p* = *0.10; r* = *-0.31, p* = *0.18*).

Right globus pallidus results. (1) FDA score of each category: significant correlations have been observed between the right globus pallidus and the Respiration, Lips, and word intelligibility (*r* = *-0.54, p* = *0.02; r* = *-0.59, p* = *0.01; r* = *-0.52, p* = *0.03*), and mild correlations with the Laryngeal (*r* = *-0.38, p* = *0.14*). (2) Acoustic features: meanwhile, there were significant correlations between the right globus pallidus and the Jaw distance, VSA (*r* = *0.54, p* = *0.01; r* = *0.50, p* = *0.02*). (3) Behavioral tasks: there were only mild correlations observed between the right globus pallidus and the SF_T1 and T3 windows (*r* = *-0.19, p* = *0.41; r* = *-0.20, p* = *0.37*).

### 3.4 The lesion-behavioral-acoustic results of thalamus

**Figure 8:**
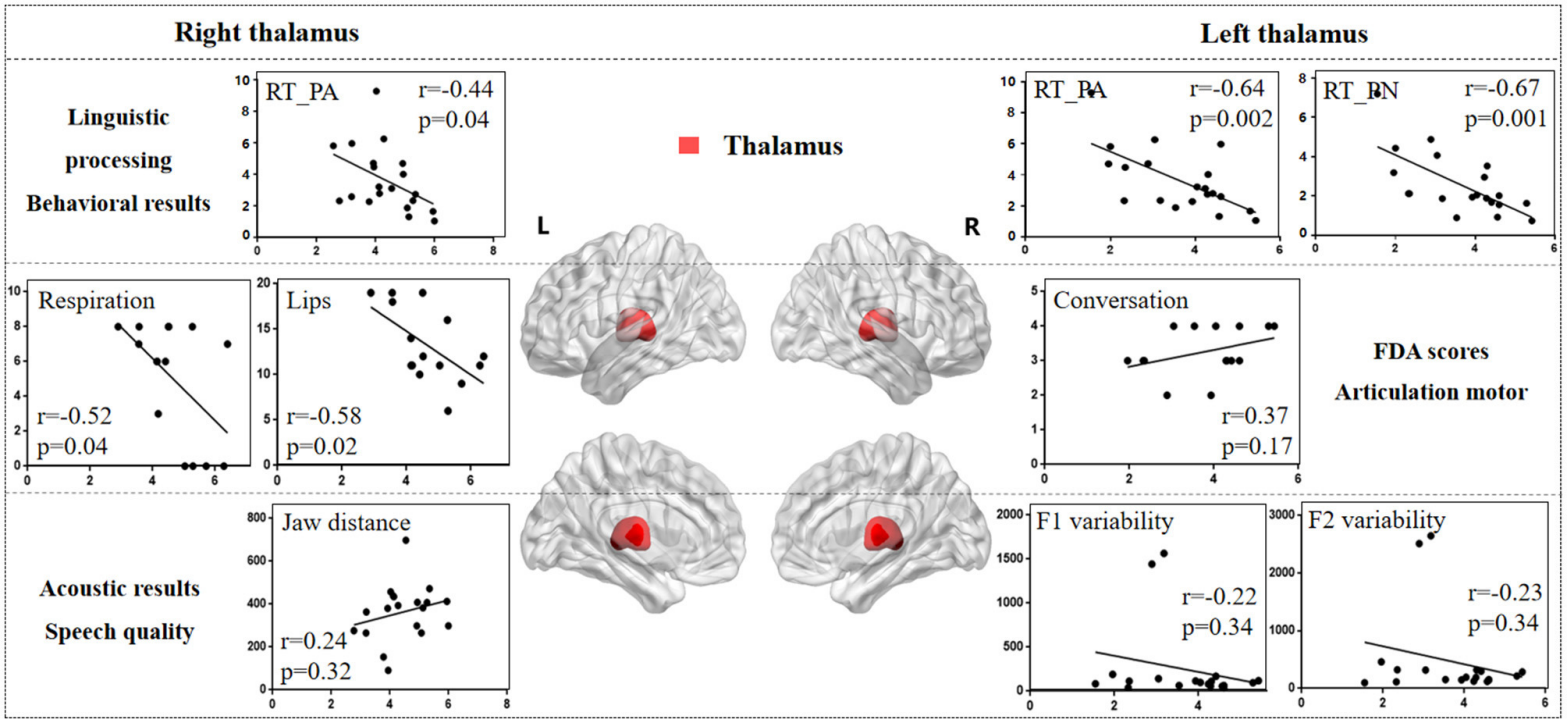
The correlation results between thalamus and behavioral tasks, FDA scores, and acoustic features. The x-axis represents the gray matter volume (VGM) of the left or right thalamus, while the y-axis displays the values of various behavioral and acoustic parameters, as well as the FDA scores.

Figure 8 illustrates the correlation results between the thalamus and various aspects of speech production. The detailed thalamus results are presented in the Supplementary material.

Left thalamus results. (1) Behavioral tasks: There were significant correlations between left thalamus and SF_T2 and T3 windows (*r* = *0.54, p* = *0.01; r* = *0.45, p* = *0.04*), PA and PN tasks (*r* = *-0.64, p* = *0.002; r* = *-0.67, p* = *0.001*), and mild correlations with SF_T4 window and CN task (*r* = *0.16, p* = *0.48; r* = *-0.36, p* = *0.11*). (2) FDA score of each category: There were only mild correlations observed between damage to the left thalamus and the Laryngeal, conversation intelligibility, and speed of intelligibility (*r* = *0.30, p* = *0.26; r* = *-0.24, p* = *0.38; r* = *0.37, p* = *0.17*). (3) Acoustic features: There were only mild correlations between left thalamus and vowel duration, F1/F2 variability, and shimmer (*r* = *-0.21, p* = *0.37; r* = *-0.22, p* = *0.34; r* = *-0.23, p* = *0.34; r* = *0.36, p* = *0.15*).

Right thalamus results. (1) FDA score of each category: significant correlations have been observed between right thalamus and the Respiration, Lips (*r* = *-0.52, p* = *0.04; r* = *-0.58, p* = *0.02*), and moderate correlations with the word intelligibility (*r* = *-0.35, p* = *0.19*). (2) Behavioral tasks: There were significant correlations observed between right thalamus and the PA, CN tasks (*r* = *-0.44, p* = *0.04; r* = *-0.42, p* = *0.06*), and moderate correlations with SF_T1, T2, T3 windows, and PN task (*r* = *0.32, p* = *0.15; r* = *0.37, p* = *0.10; r* = *0.38, p* = *0.09; r* = *-0.33, p* = *0.14*). (3) Acoustic features: Meanwhile, there were only mild correlations between the right thalamus and the Jaw distance (*r* = *0.24, p* = *0.32*).

## 4 Discussion

Seeing the shortcomings associated with the impact of different diseases and lesion locations in previous studies on dysarthria, the present study is the first to integrate multimodal neuroimaging, behavioral, and speech data to explore the direct effects of various subcortical nuclei on speech production (Polikoff and Bunnell, 1999; Kim et al., 2008). The present investigation uniquely focused on subacute stroke patients suffering from lesions in the basal ganglia and/or thalamus, with the goal of thoroughly uncovering the functions of these distinct nuclei throughout the linguistic processing and articulation stages. Linguistic processing encompassed a spectrum of functional stages as outlined by the lexical access model, and the articulation stage includes motor control of the vocal organs and acoustic features (Wilson et al., 2009).

Recent studies suggested that the basal ganglia are involved not only in motor execution, but also in the linguistic processing (Lim et al., 2014; Silveri, 2021). The present behavioral results confirmed this hypothesis and revealed the relationship between the basal ganglia and the early stages of speech production. The behavioral tasks used in present study collectively covered the full spectrum of stages of speech production, from conceptual preparation to articulation (Levelt, 1992). When task complexity escalated, both response time and error rate increased (as depicted in Figure 4, Supplementary Table A2), suggesting a heightened reliance on top-down and cognitive processing mechanisms (Wilson et al., 2009). For example, the SF task appeared to require not only phonetic encoding and articulation, but also a more substantial engagement in the conceptual preparation and word retrieval phases (Levelt et al., 1999). SF results revealed significant discrepancies, particularly in the T1 and T2 windows (as shown in Figure 4A). This indicates that dysarthria may impede speech fluency, linked to the initiation and pace of speech production, and potentially associated with the feedforward control system outlined in the DIVA model (Kearney and Guenther, 2019).

Previous research has highlighted the crucial roles of the basal ganglia and thalamus in articulation movement (Lim et al., 2014; Ziegler and Ackermann, 2017). Consistent with this, the present study found that stroke patients with basal ganglia and thalamus lesions exhibited prolonged vowel durations, reduced stability, smaller vowel space area, and decreased movement distance during speech production (see Figure 3). These findings suggest that basal ganglia and thalamic damage impairs motor control, leading to less precise and more irregular speech patterns compared to healthy adults (Mou et al., 2018). Additionally, correlation analysis revealed that different basal ganglia nuclei facilitate the motor execution of articulation stages by controlling and coordinating vocal organ movements (Silveri, 2021). The left caudate nucleus primarily influences tongue movement, while the right caudate nucleus more directly affects the motor functions of the tongue, larynx, and lips, resulting in poorer speech intelligibility. Similarly, the right globus pallidus is linked to motor abilities of the vocal organs, including lips, larynx, tongue, and respiration. These findings consistent with acoustic results, where the right globus pallidus is associated with jaw distance and VSA.

Studies on Parkinson's disease (PD) patients have shown a decline in cognitive and motor functions due to basal ganglia and thalamus atrophy (Federico et al., 2015; Tang et al., 2018). However, in stroke patients, damage in the basal ganglia and thalamus more directly affects movement of vocal organs. Exploring speech impairments caused by different diseases (e.g., PD and stroke) helps contribute to the development of targeted diagnostic and therapeutic strategies. While previous studies have linked the basal ganglia to motor functions (Lim et al., 2014; Sharma et al., 2012), our study further clarifies the correlations between different basal ganglia nuclei and motor functions of the vocal organs. Ackermann (Ackermann et al., 2014) also identified the role of the basal ganglia in executing motor programs within the feedforward system, influencing the velocity and amplitude of oral movements. Therefore, we hypothesize that, within the DIVA model, different basal ganglia nuclei contribute to varying degrees to the motor encoding and activation of each vocal organ, with the coordinated interaction between these nuclei and the thalamus playing a crucial role in the feedforward phase of speech production, including phonetic encoding, motor execution, and vocal control.

Interestingly, the present results seem to imply the presence of two distinct basal ganglia systems, or the basal ganglia is involved in two very different functions of speech production: one involved in linguistic processing and the other in articulation stage (Middleton and Strick, 2000). The right nuclei seem to primarily affect processes in motor control while the left basal ganglia are significantly associated with the linguistic aspects of speech production, such as word retrieval and selection, as well as encoding. Furthermore, the effects of different nuclei of the basal ganglia appear to be quite distinct. Prior research has identified deficits in specific aspects of speech production, including phonological decoding, lexical access, and morphological processing in individuals with extensive bilateral damage to the caudate nucleus (Pickett et al., 1998). The present findings further demonstrate that the less pronounced is the effect of damage to the left caudate nucleus during all the behavioral tasks. Specific damage to the left caudate nucleus has been linked to perseverative speech errors (Kreisler et al., 2000). In addition, in line with Robles (2005), electrical stimulation to the caudate nucleus during a picture naming task can induce perseveration, characterized by the repetition of the previous word. Damage to the left putamen also significantly influences functional processing in speech production, albeit to a lesser degree. Groenholm et al. (2015) demonstrated that both the caudate nucleus and the adjacent putamen played a role in regulating the sequencing of articulation patterns for speech sounds. Moreover, the left globus pallidus was significantly associated with the early stages of the SF task. It is hypothesized that left nuclei may primarily affect the lower-level processing stages, such as phonological retrieval and encoding, with its impact on higher-level processes potentially being an indirect result of cognitive decline, which is different from the direct cognitive impairment observed in PD patients (Lieberman et al., 1992). These insights underscore the complex and interconnected roles of the basal ganglia in the intricate processes of speech production, with lateralization and hierarchical organization being the key factors in their contributions to these functions. To achieve early diagnosis and assessment of dysarthria, it is crucial to gain a thorough understanding of the subcortical neural mechanism and its corresponding pathological features.

The thalamus is an important transmission hub for information that connects cortical regions and subcortical nuclei (Bostan and Strick, 2018; Duffy et al., 2012). The relationship between the thalamus and various behavioral tasks is notably pronounced in the left thalamus. Research indicated that thalamic damage or dysfunction often lead to speech production impairments, including articulation difficulties, fluency issues, and deficits in language comprehension (Ehlen et al., 2016). Some studies also reported that the interplay between the basal ganglia and thalamus is crucial for the initiation of movement (DeLong, 1990). Findings from behavioral tasks suggest that, as task complexity increases, the impact of the left thalamus on these tasks gradually decreases. This implies that thalamic damage could potentially lead to a deterioration in linguistic function, affecting higher-level processing such as conceptual preparation and word retrieval. Conversely, the right thalamus has a more significant influence over the control of vocal organs, including respiration and lip movements, compared to the left thalamus. Further evidence is needed to support the role of the thalamus and basal ganglia and their connection in synchronizing the timing and sequencing of speech movements, ensuring fluent and harmonious vocal expression (Ehlen et al., 2016; Schirmer, 2004).

Previous studies failed to fully elucidate the distinct effects of basal ganglia damage on cognition and linguistic processing, or investigate speech performance after accounting for the influence of cognitive factors. The present study revealed that cognitive impairments significantly impact only vowel duration and formant variability, as detailed in Supplementary Table 1. This leads to the hypothesis that dysarthria following brain injury primarily results in a reduction in the motoric capabilities of the vocal apparatus, while the associated cognitive deficits may further affect the stability and rhythm of speech. As task difficulty increased, the significance of the differences observed gradually declined across the behavioral tasks (Figure 4B). This aligns with the prior findings of Duffy et al. (2012). Cognitive capacity, as measured by the MoCA score, may predominantly influence the earlier stages of speech production, such as conceptual preparation and lexical selection. The divergent outcomes between dysarthria and cognitive impairment may also explain the inconsistent findings regarding characteristics of dysarthric speech previously reported. For example, dysarthria stemming from neurodegenerative conditions such as Parkinson's disease presents very different speech manifestations compared to post-stroke dysarthria (Silveri, 2021). Such discrepancies may arise because many studies did not consider cognition as a distinct variable, failing to clearly differentiate the effects of cognitive function (MoCA score) from those of dysarthria (FDA score), which could also confuse clinical treatment protocols for speech impairments following PD or stroke. This study is the first to integrate speech, behavioral, and neuroimaging data to explore the detailed pathological manifestations of speech production following basal ganglia or thalamic lesions. These findings may have significant implications for the development of multimodal physiological markers for the intelligent diagnosis and assessment of dysarthria.

However, several limitations are noted in the present study. First, the sample size of stroke patients is relatively small. The collection of comprehensive multimodal pathological data is challenging due to various factors, including the specific location of lesions, post-stroke complications, patient conditions, and cognitive abilities. Moreover, patients with isolated lesions are exceptionally rare and valuable. We believe that the multimodal pathological data and findings from this study, involving subacute stroke patients with basal ganglia and thalamic lesions, are highly significant for advancing the understanding of subcortical neural mechanisms underlying linguistic processing and articulatory movement. Second, the relationship between the basal ganglia, thalamus, and speech production is highly intricate, involving complex structural connectivity and functional interactions with other brain regions critical for speech processing. Future investigations will focus on subcortical structural and functional networks to provide deeper insights into the interplay between the basal ganglia, thalamus, and associated regions, as well as their distinct impacts on speech production. These issues can also provide valuable guidance for future research directions. In conclusion, this study investigated the direct impact of basal ganglia and thalamic lesions on linguistic processing and articulatory movement, contributing to a deeper understanding of the subcortical neural mechanisms underlying dysarthria. Furthermore, multimodal data encompassing neuroimaging, behavioral, and acoustic features can serve as effective indicators for developing objective evaluation methods, thereby facilitating the early diagnosis and precise intervention of speech impairments.

## 5 Conclusion

The present study utilized multi-modal mapping analyses to provide a thorough examination of the specific impacts of damage to the basal ganglia and thalamus to the various stages of speech production. It significantly advanced our understanding of the neural mechanisms associated with dysarthria. The present innovative approach not only advances our understanding of the intricate relationship between brain damage and speech production, but also offers a more nuanced perspective on the role of the basal ganglia and thalamus in this complex process. By focusing on specific dysarthria patients and integrating multiple types of data, the present findings shed light on the multifaceted nature of dysarthria and its underlying mechanisms. The research has also offered detailed insights into the functional, behavioral, and speech-related manifestations of dysarthria, which are instrumental for precise clinical diagnosis and the development of targeted treatment strategies.

## Data Availability

The original contributions presented in the study are included in the article/Supplementary material, further inquiries can be directed to the corresponding authors.
