# Supplementary material for: Mapping subcortical brain lesions, behavioral and acoustic analysis for early assessment of subacute stroke patients with dysarthria
Source: Front Neurosci. 2025 Jan 7;18:1455085. doi: 10.3389/fnins.2024.1455085 (PMC11753205; doi:10.3389/fnins.2024.1455085)
Supplement: Supplementary file 1 [file Data_Sheet_1.docx]

**
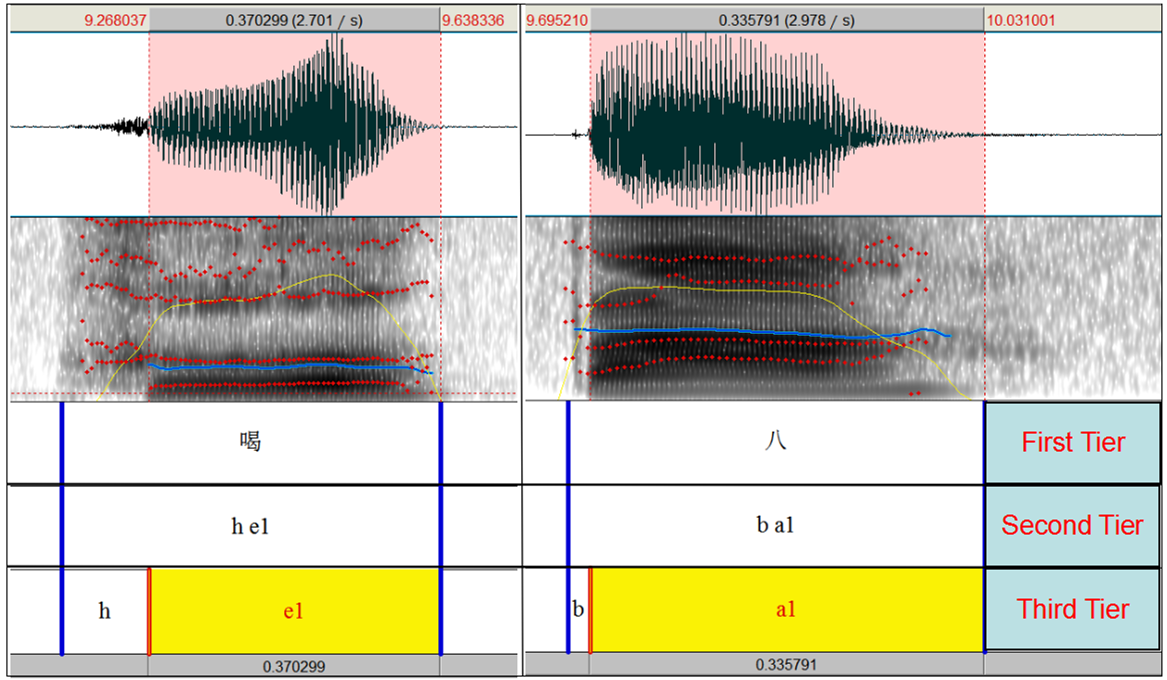
**

**Supplementary Figure 1.** The schematic diagram of vowel and consonant segments.


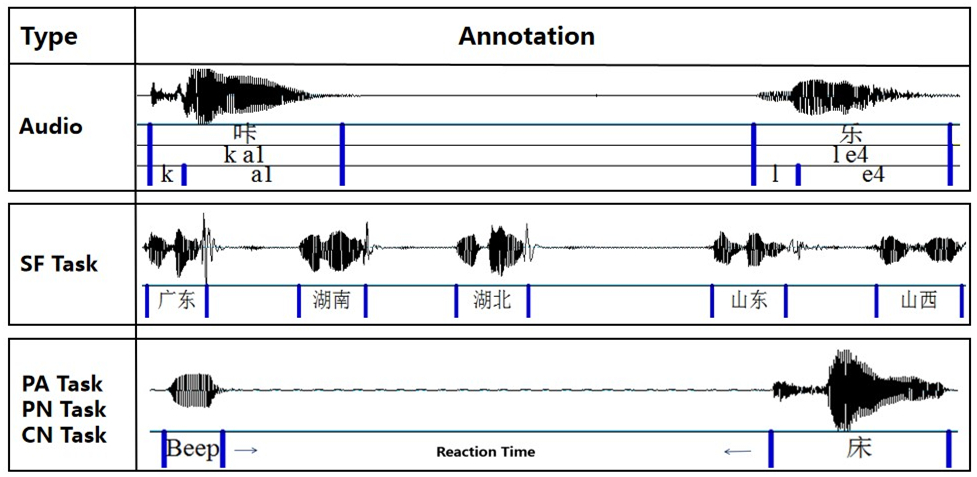


**Supplementary Figure 2.** Schematic diagram of audio and behaviour data annotation

**Supplementary Table 1.** The detailed results of acoustic parameters between the dysarthria and normal groups (*p <.05, **p <.01,***p<0.001).

| **Mean(sd)** | **Duration** | **F1 variability** | **F2** **variability** | **Jitter(a)** | **Shimmer(a)** | **HNR(a)** |
| --- | --- | --- | --- | --- | --- | --- |
| **Patient** | 0.47(0.19) | 109.05(102.51) | 220.67(198.33) | .0125(.0080) | .0747(.0409) | .9129(.0823) |
| **Normal** | 0.40(0.14) | 91.46(103.93) | 203.29(214.27) | .0105(.0121) | .0644(.0346) | .8951(.0672) |
| **Group(B/t)** | 0.11/6.81*** | -31.12/-2.74** | -201.49/-8.43*** | .00106/0.77 | .0074/1.50 | .029/2.02* |
| **MoCA(B/t)** | -0.07/-5.02*** | 16.80/1.49 | 71.30/3.03** | .0006/0.36 | .0035/0.55 | -.028/-1.50 |

| **Mean(sd)** | **Jaw distance** | **Tongue distance** | **F2i/F2u** | **VSA** | **FCR** | **VAI** |
| --- | --- | --- | --- | --- | --- | --- |
| **Patient** | 371.68(213.26) | 875.54(509.17) | 1.92(0.69) | 162593.38(137182.55) | 1.23(0.25) | 0.84(0.17) |
| **Normal** | 404.31(165.09) | 999.31(584.71) | 2.08(0.71) | 203297.53(151758.45) | 1.16(0.28) | 0.90(0.20) |
| **Group(B/t)** | 59.05/1.74 | 459.46/4.22*** | 0.46/3.43*** | 110679.95/4.60*** | -0.14/-2.91** | 0.10/3.14** |
| **MoCA(B/t)** | 1.68/0.03 | 60.74/0.44 | 0.10/0.61 | -30322/-0.93 | -0.04/-0.77 | 0.02/0.57 |

**Note**：The cognitive levels of normal or impaired were determined based on MoCA scores and education level [82]. Participants with normal cognitive level should meet the following criteria: MoCA ≥ 19/20 (education level ≤ 6 years); MoCA ≥ 22/23 (6 years ≤ education level ≤ 12 years); MoCA ≥ 24/25 (education level ＞ 12 years)

**Supplementary Table 2.** The error rate of behavioral tasks in dysarthria and normal groups (%)

| **(%)** | **PA** | **PN** | **CN** |
| --- | --- | --- | --- |
| **Patient** | 14.80 | 9.81 | 5.20 |
| **Normal** | 5.92 | 2.31 | 0.90 |

**Note:** The error rate is calculated as the number of trials with incorrect responses (including ambiguous or no responses) divided by the total number of trials.

**Supplementary Table 3.** The detailed results of behavioral features between the dysarthria and normal groups (*p <.05, **p <.01,***p<0.001).

| **Mean(sd)** | **SF_T1** | **SF_T2** | **SF_T3** | **SF_T4** | **SF_ALL** | **PA** | **PN** | **CN** |
| --- | --- | --- | --- | --- | --- | --- | --- | --- |
| **Patient** | 3.11(2.18) | 2.57(2.00) | 1.85(1.81) | 1.24(1.61) | 8.79(5.53) | 3.31(3.93) | 2.31(4.40) | 3.74(4.82) |
| **Normal** | 5.38(2.02) | 3.73(1.92) | 2.61(1.80) | 1.85(1.51) | 13.58(4.69) | 1.90(0.99) | 1.46(0.78) | 1.57(0.84) |
| **Group(B/t)** | 1.83/4.68*** | 0.76/2.11* | 0.41/1.43 | 0.31/1.21 | 3.77/3.71*** | -0.87/-3.60*** | -0.74/-3.19** | -2.65/-7.13*** |
| **MoCA(B/t)** | 1.39/2.59** | 1.13/2.29* | 1.21/2.92** | 0.95/2.53** | 3.21/2.39* | -0.25(-0.60) | -0.77/-1.48 | -0.45/0.99 |

**Note:** the parameter of SF task is the effective word number in each time window, and the parameter of PA/PN/CN tasks is the reaction time of each trial (s).

**Supplementary Table 4.** The correlation results between the basal ganglia, thalamus, and behavioral tasks.

| **r(p)** | **SF_T1** | **SF_T2** | **SF_T3** | **SF_T4** | **PA** | **PN** | **CN** |
| --- | --- | --- | --- | --- | --- | --- | --- |
| **L-caudate** | 0.15(0.50) | **0.40(0.07)** | **0.30(0.19)** | 0.03(0.89) | **-0.39(0.08)** | **-0.50(0.02)** | -0.10(0.65) |
| **R-caudate** | 0.11(0.61) | 0.19(0.40) | 0.14(0.55) | -0.15(0.50) | -0.19(0.41) | -0.38(0.09) | -0.02(0.91) |
| **L-putamen** | -0.12(0.61) | **0.47(0.03)** | 0.38(0.09) | 0.01(0.95) | **-0.56(0.008)** | **-0.59(0.005)** | -0.21(0.37) |
| **R-putamen** | -0.04(0.86) | 0.18(0.43) | 0.09(0.68) | -0.17(0.44) | -0.24(0.30) | -0.21(0.36) | -0.08(0.73) |
| **L-pallidum** | **-0.45(0.04)** | -0.33(0.15) | -0.42(0.06) | -0.22(0.34) | 0.01(0.95) | 0.23(0.31) | 0.07(0.74) |
| **R-pallidum** | -0.19(0.41) | -0.13(0.57) | -0.20(0.37) | -0.17(0.47) | -0.08(0.72) | 0.10(0.66) | 0.04(0.86) |
| **L-thalamus** | 0.13(0.56) | **0.54(0.01)** | **0.45(0.04)** | 0.16(0.48) | **-0.64(0.002)** | **-0.67(0.001)** | -0.36(0.11) |
| **R-thalamus** | 0.32(0.15) | 0.37(0.10) | 0.38(0.09) | 0.25(0.27) | **-0.44(0.04)** | -0.33(0.14) | **-0.42(0.06)** |

**Supplementary Table 5.** The correlation results between the basal ganglia, thalamus, and FDA scores.

| **r(p)** | **Reflex** | **Respira-**  **tion** | **Lips** | **Jaw** | **Laryngeal** | **Tongue** | **Intelligibility** | | | |
| --- | --- | --- | --- | --- | --- | --- | --- | --- | --- | --- |
|  |  |  |  |  |  |  | **word** | **sentence** | **conversation** | **speed** |
| **L-caudate** | -0.01(0.96) | -0.008(0.97) | -0.07(0.77) | -0.25(0.3) | 0.24(0.35) | **0.56(0.02)** | 0.15(0.56) | -0.03(0.89) | 0.12(0.63) | 0.22(0.40) |
| **R-caudate** | 0.09(0.72) | 0.24(0.35) | **0.46(0.06)** | 0.02(0.93) | **0.63(0.008)** | **0.59(0.01)** | **0.61(0.01)** | **0.42(0.10)** | **0.42(0.10)** | **0.56(0.02)** |
| **L-putamen** | -0.15(0.57) | 0.10(0.69) | -0.03(0.89) | -0.06(0.80) | 0.31(0.23) | 0.31(0.23) | 0.16(0.54) | -0.16(0.5) | 0.20(0.43) | 0.18(0.48) |
| **R-putamen** | 0.15(0.56) | -0.30(0.24) | -0.07(0.77) | -0.23(0.37) | **0.45(0.07)** | 0.33(0.20) | 0.11(0.66) | 0.03(0.90) | **0.46(0.07)** | 0.38(0.14) |
| **L-pallidum** | 0.03(0.90) | **-0.48(0.05)** | **-0.68(0.003)** | -0.32(0.22) | **-0.63(0.008)** | **-0.60(0.01)** | -**0.78(0.000)** | -**0.52(0.03)** | **-0.52(0.03)** | **-0.59(0.01)** |
| **R-pallidum** | 0.05(.83) | **-0.54(0.02)** | **-0.59(0.01)** | -0.06(0.80) | -0.38(0.14) | -0.11(0.66) | **-0.52(0.03)** | -0.09(0.72) | -0.08(0.76) | -0.31(0.23) |
| **L-thalamus** | -0.12(0.65) | -0.01(0.95) | -0.13(0.63) | 0.20(0.47) | 0.30(0.26) | 0.06(0.80) | 0.01(0.96) | -0.24(0.38) | 0.37(0.17) | 0.13(0.64) |
| **R-thalamus** | 0.00(0.97) | **-0.52(0.04)** | **-0.58(0.02)** | -0.26(0.34) | 0.05(0.84) | 0.00(0.97) | -0.35(0.19) | -0.17(0.53) | 0.35(0.19) | 0.08(0.77) |

**Supplementary Table 6.** The correlation results between the basal ganglia, thalamus, and acoustic features.

| **r(p)** | **Duration** | **F1 variability** | **F2 variability** | **Jitter** | **Shimmer** | **HNR** |
| --- | --- | --- | --- | --- | --- | --- |
| **L-caudate** | -0.22(0.35) | -0.23(0.34) | -0.20(0.39) | 0.18(0.48) | 0.19(0.44) | -0.11(0.66) |
| **R-caudate** | -0.22(0.36) | -0.25(0.29) | -0.24(0.30) | -012(0.64) | -0.09(0.70) | 0.12(0.63) |
| **L-putamen** | -0.24(0.32) | -0.27(0.26) | -0.27(0.25) | 0.06(0.79) | 0.15(0.54) | -0.20(0.42) |
| **R-putamen** | -0.01(0.95) | -0.04(0.86) | -0.03(0.90) | -0.29(0.25) | -0.40(0.10) | 0.20(0.41) |
| **L-pallidum** | 0.30(0.21) | 0.31(0.19) | 0.30(0.20) | 0.05(0.83) | -0.29(0.24) | 0.16(0.52) |
| **R-pallidum** | 0.07(0.76) | 0.09(0.71) | 0.08(0.74) | -0.20(0.43) | -0.24(0.35) | 0.14(0.56) |
| **L-thalamus** | -0.21(0.37) | -0.22(0.34) | -0.23(0.34) | -0.00(0.99) | 0.36(0.15) | -0.15(0.54) |
| **R-thalamus** | 0.06(0.77) | 0.09(0.71) | 0.10(0.67) | -0.06(0.80) | 0.09(0.70) | -0.00(0.96) |

| **r(p)** | **Jaw distance** | **Tongue distance** | **F2i/F2u** | **VSA** | **FCR** | **VAI** |
| --- | --- | --- | --- | --- | --- | --- |
| **L-caudate** | 0.08(0.71) | -0.01(0.95) | -0.01(0.94) | -0.05(0.82) | 0.005(0.98) | 0.01(0.95) |
| **R-caudate** | 0.14(0.55) | **0.47(0.04)** | 0.37(0.11) | 0.26(0.27) | **-0.44(0.05)** | 0.38(0.10) |
| **L-putamen** | 0.28(0.24) | 0.07(0.74) | 0.06(0.78) | 0.20(0.40) | -0.13(0.57) | 0.15(0.53) |
| **R-putamen** | 0.36(0.12) | 0.12(0.61) | 0.08(0.73) | 0.27(0.26) | -0.15(0.51) | 0.14(0.56) |
| **L-pallidum** | 0.17(0.47) | -0.34(0.14) | -0.31(0.18) | 0.08(0.72) | 0.37(0.10) | -0.31(0.18) |
| **R-pallidum** | **0.54(0.01)** | 0.05(0.80) | 0.05(0.83) | **0.50(0.02)** | -0.08(0.71) | 0.10(0.67) |
| **L-thalamus** | 0.21(0.38) | -0.02(0.91) | 0.004(0.98) | 0.004(0.84) | -0.09(0.68) | 0.11(0.63) |
| **R-thalamus** | 0.24(0.32) | -0.13(0.58) | -0.11(0.63) | 0.01(0.95) | 0.07(0.77) | -0.04(0.84) |

**Supplementary Table 7.** The distribution and proportion of subcortical lesion

|  | Caudate | | Putamen | | Pallidum | | Thalamus | |
| --- | --- | --- | --- | --- | --- | --- | --- | --- |
|  | Left | Right | Left | Right | Left | Right | Left | Right |
| Proportion (%) | 25% | 30% | 30% | 25% | 35% | 45% | 40% | 35% |
| Number (all) | 7 (35%) | | 8 (40%) | | 14 (70%) | | 10 (50%) | |
